# Detection of cefiderocol and aztreonam/avibactam resistance in epidemic *Escherichia coli* ST-361 carrying *bla*_NDM-5_ and *bla*_KPC-3_ from foreign fighters evacuated from Ukraine

**DOI:** 10.1128/aac.01090-24

**Published:** 2024-09-20

**Authors:** Melissa J. Martin, Ting L. Luo, Valentyn Kovalchuk, Viacheslav Kondratiuk, Henry D. Dao, Iryna Kovalenko, Brandon J. Plaza, Joanna M. Kettlewell, Cole P. Anderson, Jason R. Smedberg, Ana C. Ong, Yoon I. Kwak, Joshua S. Hawley-Molloy, Jason W. Bennett, Patrick T. McGann, Francois Lebreton

**Affiliations:** 1 Multidrug-Resistant Organism Repository and Surveillance Network (MRSN), Walter Reed Army Institute of Research, Silver Spring, Maryland, USA; 2 Department of Microbiology, National Pirogov Memorial Medical University, Vinnytsia, Ukraine; 3 Department of Emergency and Military Medicine, National Pirogov Memorial Medical University, Vinnytsia, Ukraine; 4 Department of Pathology, Landstuhl Regional Medical Center, Landstuhl, Germany; 5 Division of Medicine, Landstuhl Regional Medical Center, Landstuhl, Germany; Johns Hopkins University School of Medicine, Baltimore, Maryland, USA

**Keywords:** Ukraine, antibiotic resistance, carbapenem resistance, genomic surveillance

## Abstract

Genomic surveillance detected clonal *Escherichia coli* sequence type-361 isolates carrying *bla*_NDM-5_, *bla*_KPC-3_, *bla*_CTX-M-15_, and *rmtB1* from a patient in Ukraine and four wounded foreign soldiers evacuated to Germany. Isolates were non-susceptible to carbapenems, aminoglycosides, and cefiderocol and aztreonam/avibactam due to a PBP3 YRIN insertion and the *bla*_CMY-145_ AmpC β-lactamase. Coordinated surveillance efforts across civilian, military, and veteran healthcare systems are essential to prevent further spread as international volunteers return home after medical evacuation from Ukraine.

## INTRODUCTION

Carbapenem-resistant Enterobacterales (CRE) are considered as critical priority pathogens by the WHO (1). CREs mainly result from the production of carbapenem-hydrolyzing enzymes such as serine carbapenemases (e.g., KPCs and OXA-48) or metallo-β-lactamases (MBLs) (2). MBLs are of particular concern as most clinically available β-lactams/β-lactamase inhibitor (BL/BLI) combinations, the main treatment for CREs, are not active (3). For these strains, polymyxins are one of the last treatment options available, along with the new siderophore cephalosporin antibiotic, cefiderocol or aztreonam/avibactam, a BL/BLI combination that shows excellent activity against MBL producers (4).

Extensively drug-resistant (XDR) *Escherichia coli* sequence type (ST)-361 carrying the *bla*_NDM-5_ MBL has recently been reported in multiple European countries (5–7), including in isolates resistant to cefiderocol (6). Here, between May 2023 and January 2024, five *E. coli* ST-361 co-carrying *bla*_NDM-5_ and *bla*_KPC-3_ were cultured from a war wound of a hospitalized patient in Ukraine and peri-rectal swabs of four international soldiers wounded in Ukraine and evacuated to a US military treatment facility (MTF) in Germany.

**Fig 1:**
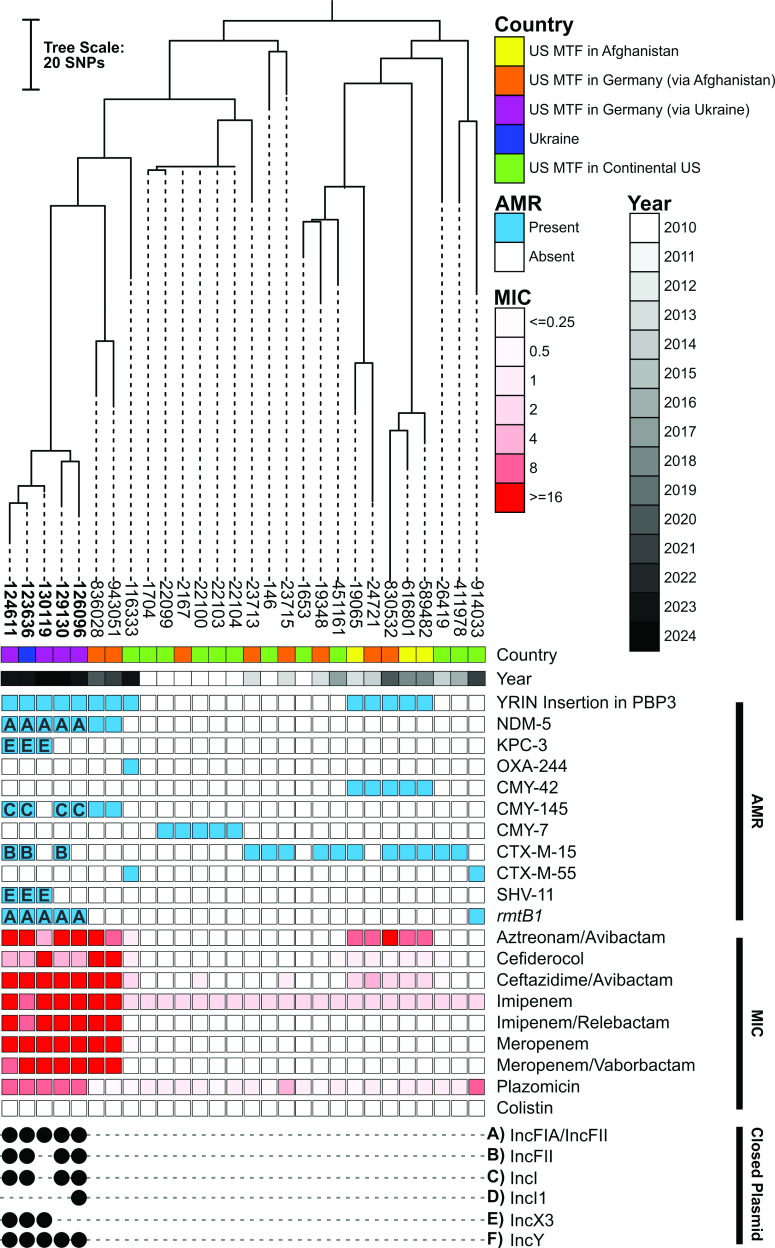
A core genome phylogenetic tree of ST-361 *E. coli* (*n* = 28) from the US Military Health System and Ukraine. Country of origin, year of collection, and presence (closed square) or absence (open square) of selected antimicrobial resistance genes and minimum inhibitory concentrations of selected antibiotics. Replicon typing (**A–F**) of closed plasmids is indicated by the closed black circle. For closed sequences (label in bold font), the genetic location of antimicrobial resistance genes is indicated by the letter of the replicon type identified in the plasmid. The midpoint was used as a root for the phylogenetic tree.

To compare, 23 historical *E. coli* ST-361 isolates were identified from the Multidrug-Resistant Organism Repository and Surveillance Network (MRSN) isolate collection. These were cultured from 23 patients between 2010 and 2022 being treated in MTFs in the continental US (*n* = 12), Germany (*n* = 8), or Afghanistan (*n* = 3) and had no known association with Ukraine (Table S1). Whole genome sequencing (Illumina) was performed on all isolates, and the five isolates linked to Ukraine (Fig. 1) were also sequenced by long-read sequencing (Oxford Nanopore Technologies), as previously described (8). Finally, to correlate genotype and phenotype, antibiotic susceptibility testing was performed using a customized Sensititre panel (Thermo Scientific) and broth microdilution for aztreonam/avibactam and cefiderocol, with breakpoints interpreted using 2023 CLSI guidelines where available. For aztreonam/avibactam, a tentative breakpoint of *S* ≤ 8 µg/mL was applied based on the recent study by Sader and colleagues (9).

Phylogenomic analysis of all 28 ST-361 isolates revealed that none of the 18 isolates collected pre-2020 carried an acquired carbapenemase compared to 8 out of 10 collected after 2020 (Fig. 1; Table S1). Despite being monophyletic and genetically related (single nucleotide polymorphism [SNP] counts ranging from 11 to 183), the eight carbapenemase producers did not harbor the same carbapenemase genes. A single isolate from the US in 2023 carried *bla*_OXA-244_ (OXA-48-like) chromosomally located within a truncated Tn*51098*, similar to previous observations (10). The remaining seven isolates carried *bla*_NDM-5_. These included five clonal isolates (11–57 SNPs), from the patients associated with Ukraine in this study, for which long-read sequencing was performed (Fig. 1; Table S1). Analysis of the complete genomes revealed these five isolates harbored an IncFIA/IncFII-type plasmid co-carrying the 16S rRNA methyltransferase gene *rmtB1* and *bla*_NDM-5_ (Fig. S1A). The latter was found on a 10.8-kb IS*26*-formed composite transposon, which also carried a typical class 1 integron containing resistance genes *sul1*, *qacE*, *aadA5*, and *dfrA17,* as previously described (Fig. S1B) (11). Interestingly, isolate 130119 uniquely carried four identical copies of the transposable element carrying *bla*_NDM-5_. Besides this exception, the IncFIA/IncFII plasmids were virtually identical (>99.9% nucleotide identity over the full length) to each other and to p1606a first detected from an ST-361 isolate in Switzerland in 2020 (5). Finally, three of the five isolates also carried a virtually identical IncX3 plasmid co-harboring *bla*_KPC-3_ and *bla*_SHV-11_ (Fig. S1C), similar to previously described p1606b (5).

Phenotypically, all *bla*_NDM-5_ carrying isolates were non-susceptible to imipenem (≥8 µg/mL), meropenem (≥16 µg/mL), imipenem/relebactam (≥8 µg/mL), meropenem/vaborbactam (≥8 µg/mL), and ceftazidime/avibactam (≥16 µg/mL) (Fig. 1; Table S1). These also had high minimum inhibitory concentrations (MICs) against aztreonam/avibactam (from 4 to ≥16 µg/mL) and cefiderocol (from 4 to 16 µg/mL) (Fig. 1; Table S1). Similar to findings from Simner and colleagues (12), the increased copy number of *bla*_NDM_ likely explained the increased cefiderocol MIC in isolate 130119 (16 µg/mL) compared to the four clonal isolates (4 µg/mL) with a single copy of the gene. Further, six isolates without *bla*_NDM-5_ also had increased MICs to cefiderocol, albeit more moderate (0.5 to 1 µg/mL). Increased cefiderocol MICs correlated exactly (13 of 13 isolates) with the presence of a four-amino acid insertion (YRIN at position 333) within PBP3, a feature previously linked to cefiderocol resistance (6). Interestingly, insertions in PBP3 also cause a reduced affinity for aztreonam resulting in a moderate to high (in association with an acquired AmpC β-lactamases) MIC increase for aztreonam/avibactam in *E. coli* (13). Here, 13 isolates had increased MIC for aztreonam/avibactam: 2 had a moderate increase (1–4 µg/mL) and only carried the PBP3 YRIN insertion and 11 had a high increase (8–16 µg/mL) and additionally carried an acquired *bla*_CMY-42_ or *bla*_CMY-145_. The latter included four of the five isolates linked to Ukraine, which carried *bla*_CMY-145_ on an IncI plasmid (Fig. 1).

In conclusion, upon admission to a US MTF in Germany, four international soldiers wounded in Ukraine were colonized with a dual-carbapenemase-producing *E. coli* with reduced cefiderocol susceptibility. The same strain was also cultured in Ukraine from a wounded Ukrainian soldier. This follows the recent detection of six distinct XDR strains/species from a single patient at the same German-based US MTF (14), which began receiving wounded foreign and US volunteers from Ukraine in June 2022. The potential spread of XDR *E. coli* ST-361, carrying multiple carbapenemases and mutations conferring resistance to last-line treatment options, is concerning. Joint surveillance efforts in civilian, active military, and veteran healthcare systems are necessary to prevent further spread as these international soldiers return home.

## Data Availability

Genomes described herein have been deposited at GenBank under BioProject PRJNA1116075.

## SUPPLEMENTAL MATERIAL

Fig. S1 aac.01090-24-s0001.docx Comparisons of plasmids.

Table S1 aac.01090-24-s0002.xlsx List of isolates and metadata.

ASM does not own the copyrights to Supplemental Material that may be linked to, or accessed through, an article. The authors have granted ASM a non-exclusive, world-wide license to publish the Supplemental Material files. Please contact the corresponding author directly for reuse.
