# Supplementary material for: Detection of cefiderocol and aztreonam/avibactam resistance in epidemic Escherichia coli ST-361 carrying blaNDM-5 and blaKPC-3 from foreign fighters evacuated from Ukraine
Source: Antimicrob Agents Chemother. 2024 Sep 20;68(11):e01090-24. doi: 10.1128/aac.01090-24 (PMC11539215; doi:10.1128/aac.01090-24)
Supplement: Fig. S1 — Comparisons of plasmids. [file aac.01090-24-s0001.docx]

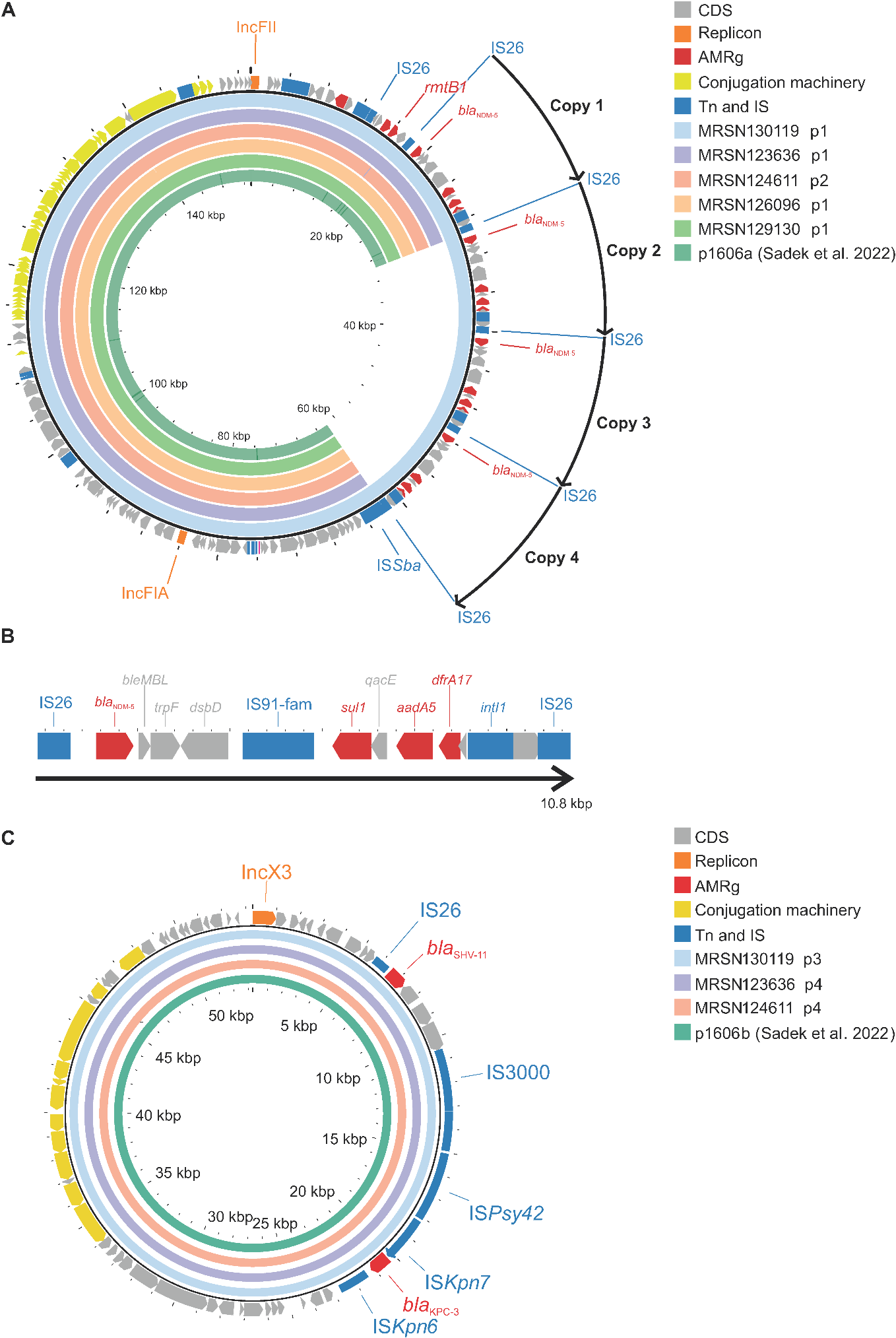


**Supplemental Figure 1**. Characterization of carbapenemase-carrying plasmids in 5 clonal ST-361 *E. coli* from the US Military Health System and Ukraine. (**A**) Comparisons of IncFIA/IncFII circularized plasmids carrying *bla*_NDM-5_ using MRSN130119_p1 as a reference. (**B**) Genetic organization of the IS*26*-formed composite transposon carrying *bla*_NDM-5_ harbored by the IncFIA/IncFII plasmids. (**C**) Comparisons of IncX3 circularized plasmids carrying *bla*_KPC-3_ using MRSN130119_p3 as a reference. Plasmids p1606a and p1606b first detected from an ST-361 isolate in Switzerland in 2020 (5) were virtually genetically identical to the IncFIA/IncFII and IncX3 plasmids, respectively.
